# Dendrimers: Amazing Platforms for Bioactive Molecule Delivery Systems

**DOI:** 10.3390/ma13030570

**Published:** 2020-01-24

**Authors:** Claudia Sandoval-Yañez, Cristian Castro Rodriguez

**Affiliations:** 1 Institute of Applied Chemical Sciences, Faculty of Engineering, Universidad Autonoma de Chile, El Llano Subercaseaux 2801, San Miguel 8910060, Santiago-Chile, Chile; 2 Departamento de Química, Facultad de Ciencias, Universidad de Tarapacá, Avenida General Velásquez 1775, Arica-Chile 1000007, Chile; cicastror@uta.cl

**Keywords:** dendrimers, bioactive molecules, drug delivery systems, cancer disease, cardiovascular disease

## Abstract

Today, dendrimers are the main nanoparticle applied to drug delivery systems. The physicochemical characteristics of dendrimers and their versatility structural modification make them attractive to applied as a platform to bioactive molecules transport. Nanoformulations based on dendrimers enhance low solubility drugs, arrival to the target tissue, drugs bioavailability, and controlled release. This review describes the latter approaches on the transport of bioactive molecules based on dendrimers. The review focus is on the last therapeutic strategies addressed by dendrimers conjugated with bioactive molecules. A brief review of the latest studies in therapies against cancer and cardiovascular diseases, as well as future projections in the area, are addressed.

## 1. Introduction

Today, most pathologies, whether chronic or not, are treated through oral, mucosal, dermal, and transdermal administration of drugs. Most of the effects of the drugs used to cure diseases have a high percentage of inhibition of the target agents that generate the pathology or significantly reduce the symptoms of it. The problem with some drugs is that they must be administered in high doses, to reach a positive therapeutic, but the higher doses usually result in unwanted side effects [1,2,3,4]. These side effects are because some drugs that have low bioavailability, so they must be administered in high doses. For this reason, in the last decades, the challenge has been focused on the investigation of the targeted drug delivery systems [5,6,7,8,9,10,11,12,13]. There is a large variety of organic systems that have been considered as drug delivery systems, such as micelles, liposomes, and polymeric nanoparticles. All these systems must meet some requirements to be applied as the drug carrier agents, namely nanoscale size, biocompatible, bioresorbable, water solubility, and monodisperse structure [13,14,15,16]. Among these synthetic nanostructures, dendrimers have been one of the most studied in the last twenty years.

**Scheme 1:**
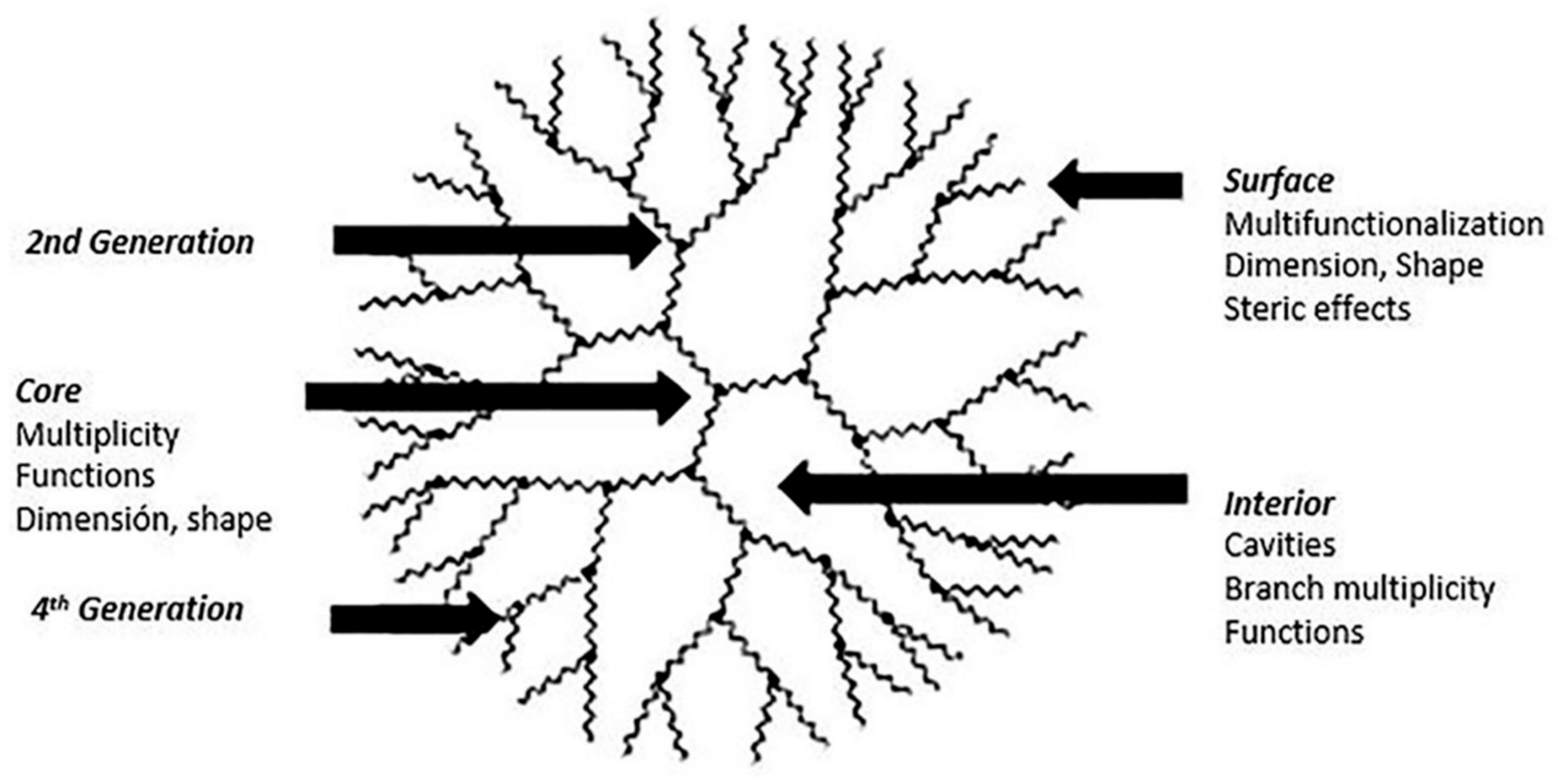
Schematic representation of a dendrimer of fourth-generation. This scheme was provided thanks to Reference [20].

Dendrimers are very attractive macromolecules, both from the challenge proposed by their design and chemical synthesis and from the range of applications they have given in different areas. They are three-dimensional structures highly branched and radially symmetrical (Scheme 1). They have three very well defined structural regions, a central junction structure (core) with multiple internal repeating units covalently linked to the nucleus (called generations, G), and finally, a terminal chemical structure that forms the multifunctional surface of a dendrimer [17]. These attractive macromolecular architectures were reported in the late 70’s by Vögtle et al. and have been the subject of numerous studies since then. In later years Tomalia, Newkome, and Frechet, independently, reported two new synthetic routes to obtain dendrimersoma [18,19]. The chemical architecture of the dendrimers overcome other polymers (linear, branched or cross-linked) since they have attributes like monodisperse structures (desirable for applications in nanomedicine), control over macromolecular growth, multifunctional surface structure, internal cavities available to host small molecules, increased solubilization, among many other advantages that enable them to be used in several fields. Nanomedicine is one of the applications in which dendrimers have been studied and tested, using as a nanocarrier bioactive molecules, imaging agents, or transfection of genes.

In the last ten years, numerous publications, over 29 thousand articles, and more than 6 thousand reviews (source: WOS, January 09–2020) have been published on a drug delivery system based on dendrimers. The present review primarily focuses on progress carried out in the last few years in dendrimers and their applications as drug delivery systems. Here, we will give an overview of the dendrimer types and their properties, and in a second part give we will present pharmacodynamic and pharmacokinetic studies which have been done regarding the transport and control release of bioactive molecules based on dendrimers. Finally, the latest studies concerning the release of drugs based on dendrimers focused on cancer and cardiovascular diseases and prospects are reported.

## 2. Dendrimers Types and Their Properties

**Scheme 2:**
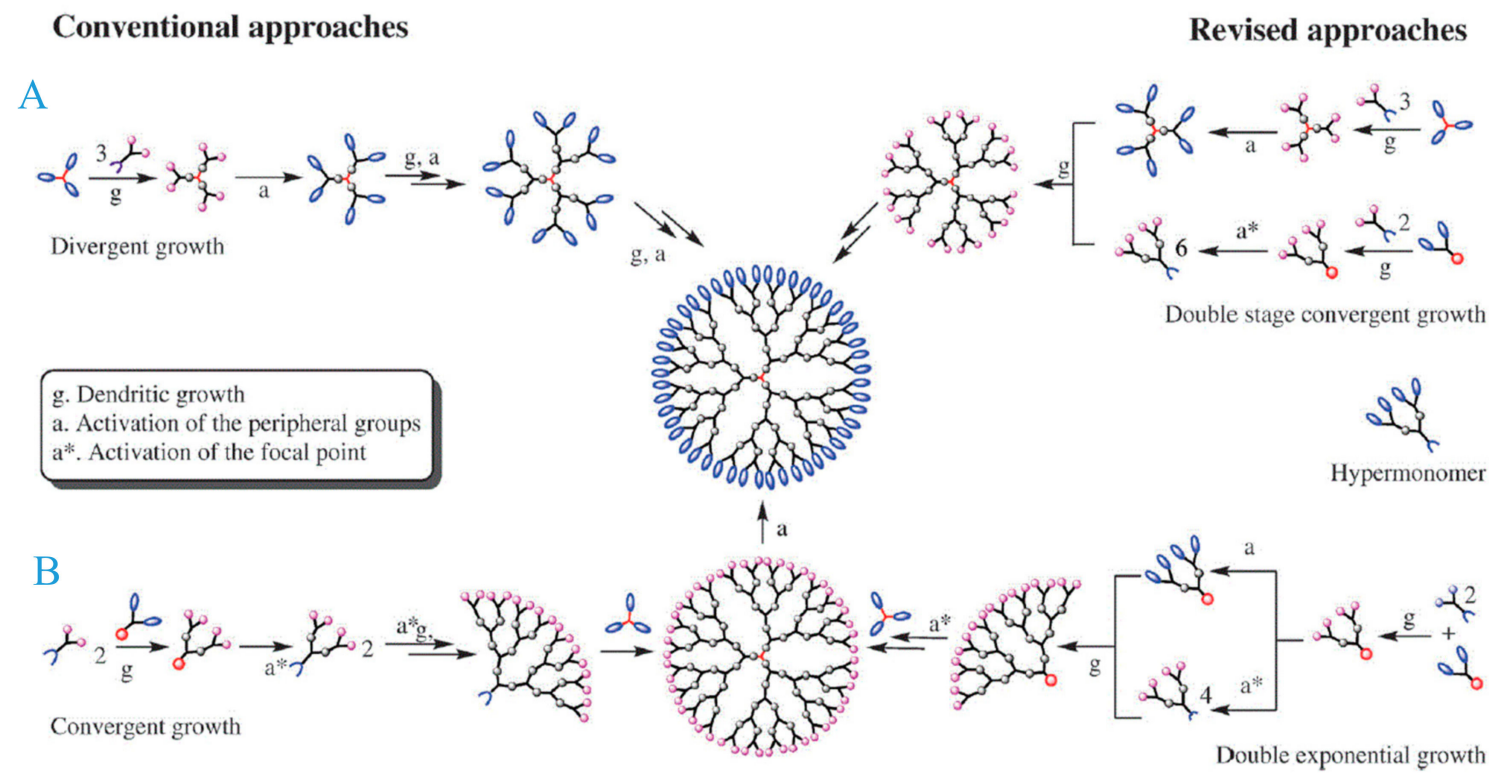
Simplified representation of dendrimers synthesis by (**A**) divergent and (**B**) convergent strategies. This scheme was provided thanks to Reference [26].

Dendrimers are three-dimensional macromolecules that have a radial architecture, which contain several branching points, and its dimensions are in the nanoscale range (Scheme 1). The chemical nature of the dendrimers can be of organic or inorganic origin with a central nucleus constitutes its morphology and, together with it, grow the so-called “generations” (G) or branches. Branches that grow and give size to the dendrimer, finally they are the groups of the periphery or terminal groups, those that have the virtue of being tunable, according to the application given to the dendrimer. The synthesis of the different types of dendrimers contemplate two mostly strategies: convergent and divergent methods. The synthesis of dendrimers through divergent method initially proposed by Vögtle [21], and subsequently developed independently by Tomalia [18] and Newkome [22,23], begins with a polyfunctional molecule that acts as a core, on which successive layers of monomer units are chemically bonded (Scheme 2a). The repetitive sequence of two reactions is used to add generations to the core, which correspond to the activation of the functional groups and their subsequent assembly with the other monomers. The advantage of the divergent method is that high molecular weight architectures can be achieved on a nanometric scale. The main disadvantage is that terminal functional groups cannot always be reacted stoichiometrically, leading to structural defects. The convergent method introduced by Hawker and Fréchet [24] involves the obtaining dendritic fragments called dendrons, through repetitive reactions, which are joined at a later stage to a central core consisting of a polyfunctional molecule (Scheme 2b). The functionality of the core determines the number of dendrons that can be bound. Convergent synthesis is normally used only to form dendritic structures of low generations, since steric hindrance limits the coupling of bulky dendrons to a core of reduced dimensions.

Recently, two new dendrimer synthesis strategies have been reported, “Lego” and “Click” chemistry [25]. The type of dendrimer required determines the type of synthesis strategy that is applied. 

### 2.1. Orthogonal Coupling or “Lego” Chemistry Strategy

Dendrimers of different chemical natures can be synthetized by this strategy, namely dendrimers containing polycarbamate/urea, phosphorus, aromatic polyamide, among others, to obtain an increase of the number of end groups, and this form high generation dendrimers [27,28]. Majoral et al. synthetized high generation dendrimers with 750 different end groups obtained in three steps [20,29]. This methodology to obtain dendrimers is advantageous since it used a minimum amount of solvent, products of facile purification, and only nitrogen and water are obtained as by-products. 

### 2.2. “Click” Chemistry Strategy

**Scheme 3:**
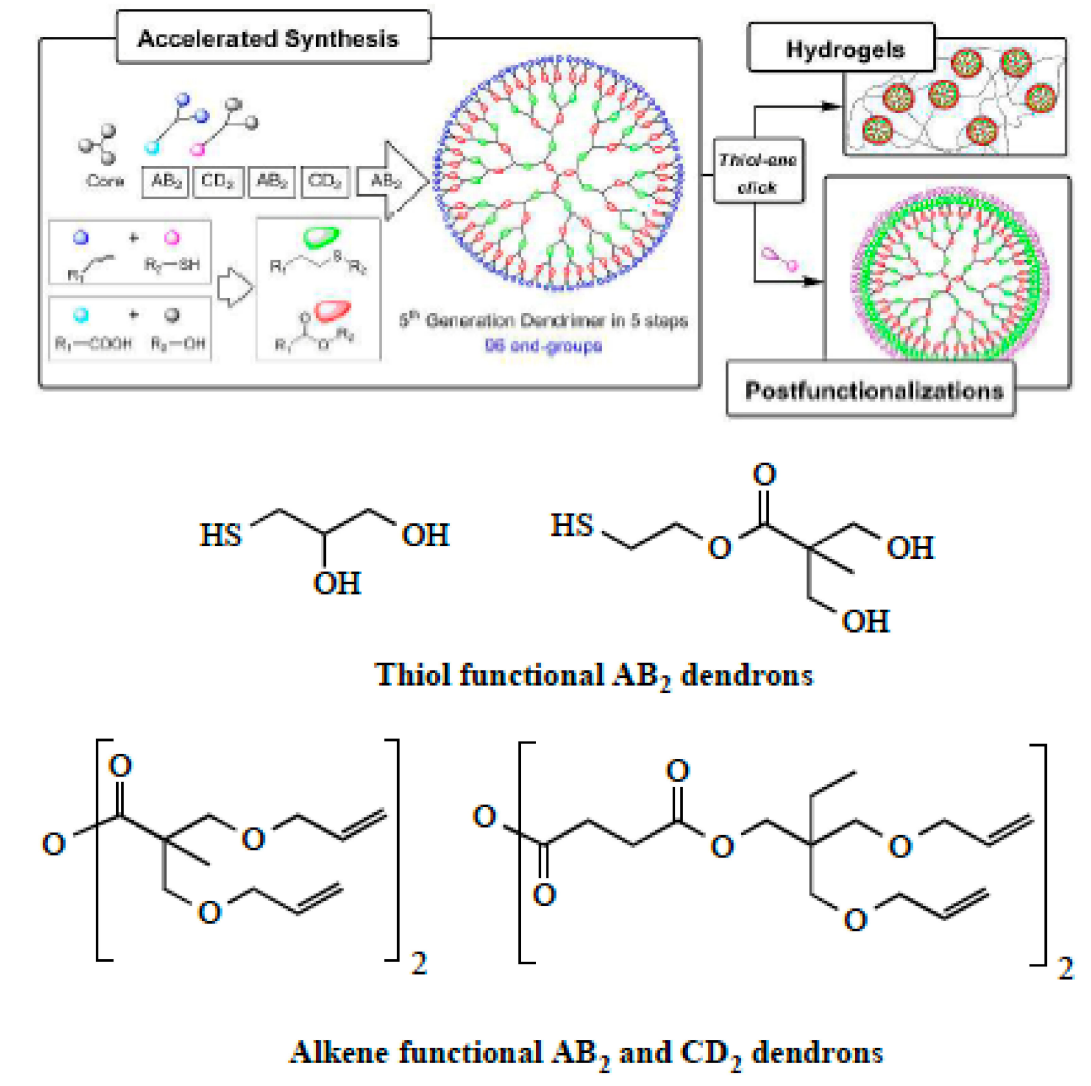
General illustration for the synthesis of dendrimers using “Click” chemistry method by of a combination of “thiol-ene” and esterification reactions. This scheme was provided thanks to Reference [33].

**Figure 1:**
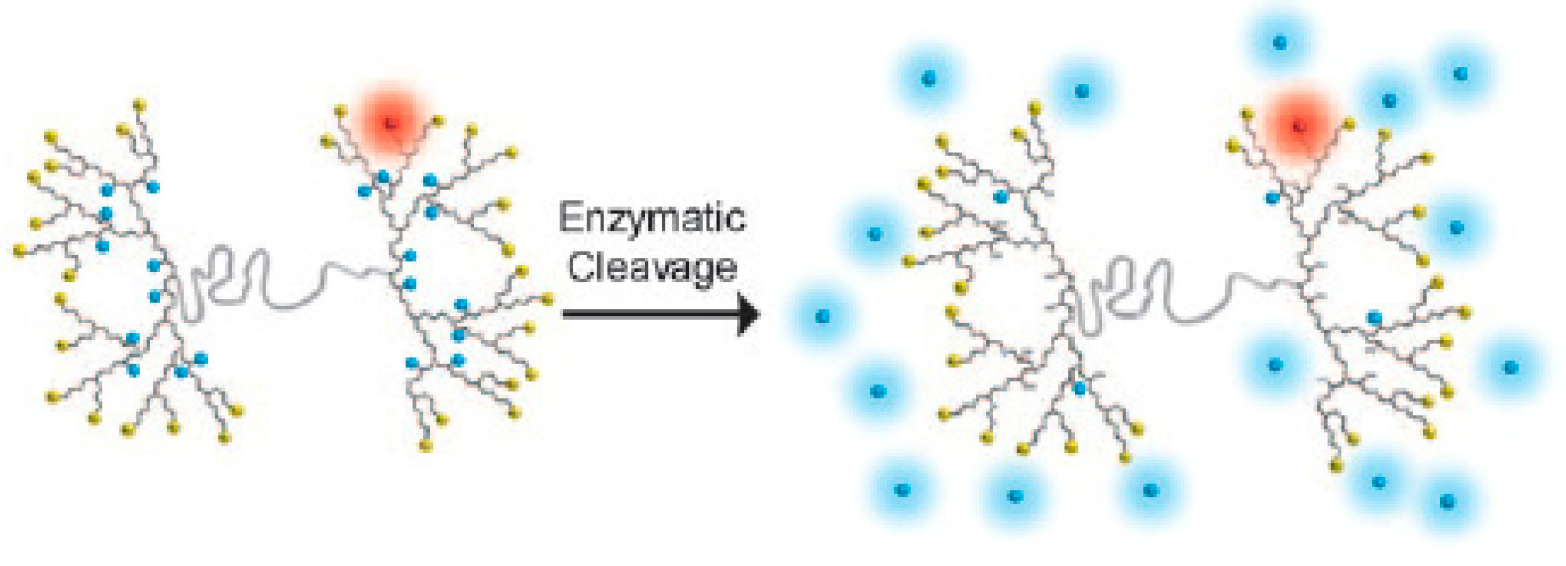
Scheme of the design of a dendritic platform, containing a dual functionalization and surface protonated amino groups (yellow). In blue: the dye internally bound to the dendritic system, through a cleavable junction by hydrolytic enzymes. In red: the cell tracking dye not cleavable by hydrolytic enzymes. This scheme was provided thanks to reference [32].

The “click” chemistry methodology allows obtaining new compounds of biological interest from highly energetic reagents through a series of highly thermodynamic and orthogonal processes. These two characteristics, high reactivity and orthogonality, allow the click-type reactions to proceed with quantitative yields and, therefore, only simple purifications are required. “Click” chemistry is a synthetic methodology remarkable which its care with the environment since it only generates as a by-product sodium chloride, mainly [30]. This reaction mechanism has been used both syntheses and modification of polymer and materials [31]. Divers types of polymers can be obtained by this strategy, specifically through called “thiol-ene” chemistry reactions. In recent years, “thiol-ene” chemistry has been used to synthesized dendrimers, star polymers and hyperbranched polymers. A comprehensive review by Lowe [31], describes the reaction mechanisms of “thiol-yne” click coupling chemistry, and their applications in dendrimers synthesis using different combinations of AB2 and CD2 monomers (see Scheme 3). In the same way, Amir et al. [32] carried out a “click” strategy to obtain fourth-generation dendritic scaffold with two dyes attach internally with coumarin units through a cleavable linker and on superficial region with Alexa647 dye. In addition, some protonated amino groups remained on the surface of the dendritic system, which allows the internalization of the system within the cells. This strategy allowed the monitoring of the dendritic scaffold within the cellular environment, and the appearance of fluorescence could also be observed, due to the release of coumarin dye to the hydrolytic enzyme cleavage inside the cell (Figure 1). This type of thiol-yne and thiol-epoxy synthesis reaction strategy allows versatility to obtain dendritic platforms for transport and release of drugs to target tissues.

Dendrimers have attractive properties since it offers great advisability for the storage, transport, and controlled release of bioactive molecules. A triazole-base dendrimer contained bile acid surface groups was synthesized by “click” chemistry strategy [34]. In addition, “Janus” dendrimers and “onion peel” were obtained using this strategy [35]. Pérez-Campos et al. has synthesized two different dendrimeric structures and the precursor dendron with aimed to evaluate their role in the antichagasic activity as drug carriers and as prodrugs. The dendrons were derived from ethynylestradiol molecules modified with PAMAM type fragments through a “click” reaction to produce a triazolic ring [36].

Table 1 gives a list of different types of dendrimers obtained with the synthetic strategies described above and the authors who have reported these works. Moreover, the reader can go to a review completely dedicated to the different methods of dendrimer synthesis, recently published by Lyu et al. [37]

## 3. Dendrimers Properties

### 3.1. Monodispersity

Dendrimers are monodispersed macromolecular architectures, unlike to linear polymers, this is an important property to be applied in nanomedicine. High-performance liquid chromatography (HPLC), size exclusion chromatography (SEC), mass spectrometry (MS), gel electrophoresis, and transmission electron microscopy (TEM) techniques have been extensively using to verify the monodispersity of dendrimers [55,56]. The advances in new synthetic strategies have allowed obtaining better yields in monodisperse dendrimers [57]. Recently, Malkoch et al. [58] presented a simple post-functionalization approach of monodisperse dendrimers of bis-MPA from generation one to five. Two types of esterification reactions were used to carry out the synthesis: (a) activation of BOC-protected β-alanine through anhydride coupling, (b) fluoride-promoted esterification with activation by carbonyl diimidazole. This last reaction had a substantial improvement in product purity, and scalability. 

### 3.2. Nano-Size

**Scheme 4:**
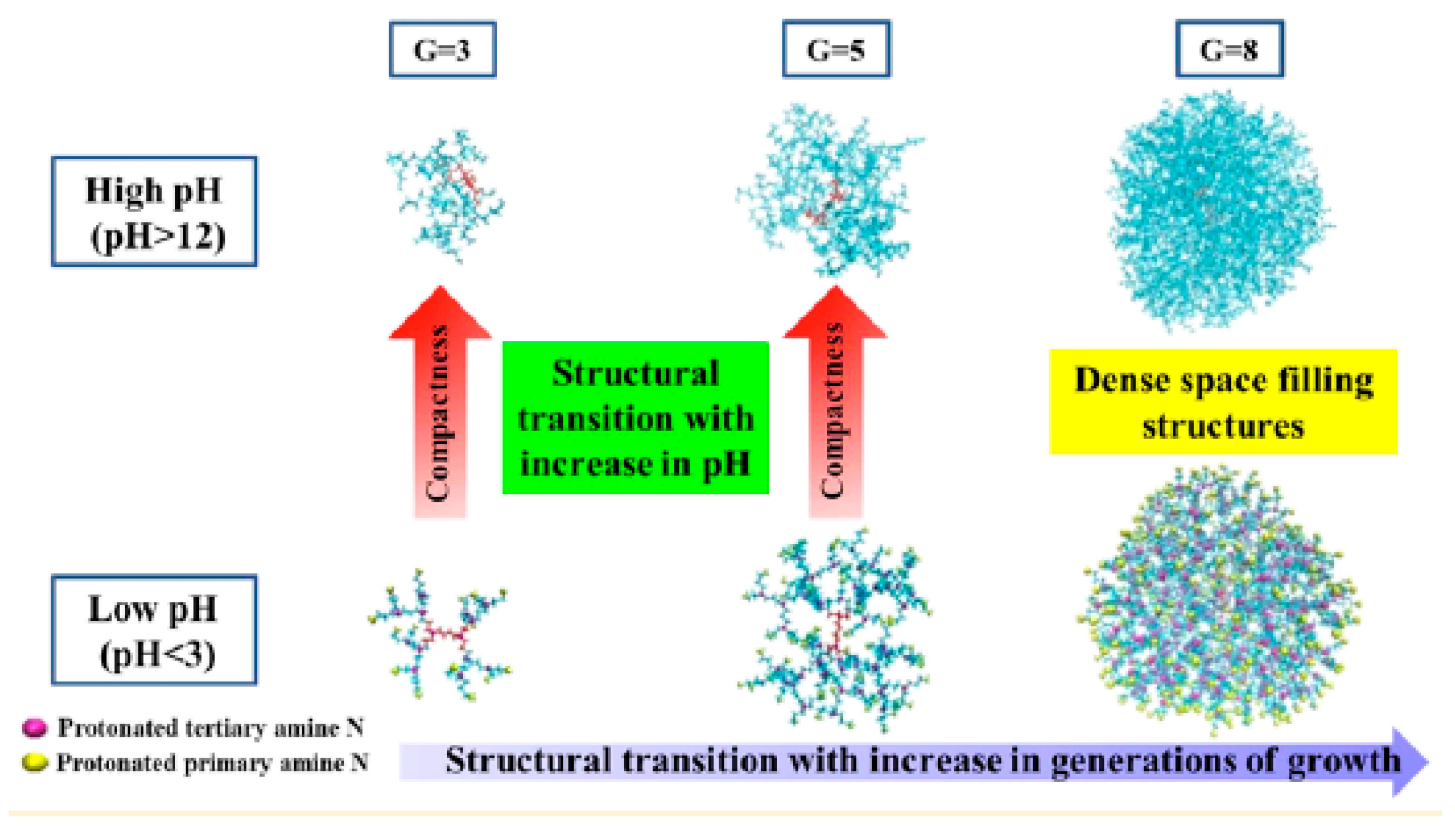
Illustration of the variation of the dendrimer’s conformation with respect to pH value and their generation. This scheme was provided thanks to Reference [64].

Regarding the size of dendrimers, these have a nanoscale dimension, and can be considered similar to other biomacromolecules such as proteins, which makes them very attractive from that point of view [59,60]. The size of a dendrimer is determined by the number of generation. Therefore, it is easy to think that if the number of generations increases, the size of the dendrimer will also do so [60,61,62]. Polyamidoamide (PAMAM) an important class of commercial dendrimers, where the diameter of the generations 1–10 increases from 1.1 to 12.4 nm [27,63]. A recent study carried out by Biswas et al. [64] on the effect of pH on the size and conformational structure of Poly(propylene imine) dendrimers through molecular dynamics simulation, shows that the size of dendrimers increase according to decreasing of pH for all generations (G3 to G8) (Scheme 4). This behavior may be due to the electrostatic repulsions between the protonated primary and tertiary amine groups at neutral and low pH, the degree of back-folding decrease with the decreasing in the pH [65,66]. These types of studies are important since they can be used to monitor and interpret the conformational behavior of dendrimers as a function of pH, for several applications such as drug delivery systems. In addition, Diaz et al. [67] has determine that there is a direct relationship between the size of the dendrimer and its circulation time in the bloodstream. The functionalization of the end-groups in the outer surface of dendrimers with different functional groups modify their physicochemical properties and biological behaviors [68,69]. Recently, dendrimers decorated with positively charged amino acids on the superficial were synthesized [70]. Recently, Govender et al. [71] formulated and evaluated novel pH-responsive lipid-dendrimer hybrid nanoparticles for the targeted delivery of vancomycin in the site infection. The study has shown that the pH-responsive release of nanoparticle enhanced antibacterial efficacy and intracellular delivery as antibiotic. 

### 3.3. Physicochemical Properties 

In recent years, numerous studies about the physicochemical properties of dendrimers have been carried out since these have several outstanding physicochemical properties, which make them synthetic macromolecules with a wide range of applications [17,72,73]. Dendrimers have multifunctional surfaces that give the possibility of modifying their solubility and interactions with other molecules in different environments. An adequate solubility has been one of the problems when selecting some bioactive molecules for pharmaceutical product development. Drugs with low solubility in the bloodstream determine the pharmaceutical and therapeutic performance, so dendrimers are an excellent alternative to enhance a low solubility drug [74,75]. Many compounds that are excellent candidates as potent drugs against some pathologies do not continue with more advanced studies, due to their limited aqueous solubility. In recent years, several researches have been focused to solubilize powerful hydrophobic drugs to increase their bioavailability [76]. Some research has been conducted on the study of physicochemical parameters such as, water solubility, Log*P*, polar surface area, and the number of hydrogen bond acceptor and hydrogen bond donors as a measurement to elucidate the drug molecules solubility grade [77]. The modification of the surface of dendrimers offers a great opportunity to improve not only the solubility of drug-like molecules but also to enhance the biocompatibility and permeability in a cellular environment, i.e., improve their physicochemical properties. The polycationic periphery of the dendrimer promotes interactions with lipids of the cell membrane, enhancing drug solubility. The nanoformulations, such as prodrugs based on dendrimers modification have been intensively studied to improve the bioavailability and solubility of drugs [78]. Highly hydrophobic drugs incorporated into polycationic dendrimers through physical through electrostatic interactions and hydrogen bonding can be obtained. Pentacyclic triterpenoid acids encapsulated into the G4, and G5 amino acids-modified PAMAM dendrimers have been prepared and solubilized, allowing a better bioavailability, and an increased pharmacokinetic [79]. The different interactions present between dendrimers and drugs, namely electrostatic interactions, dispersion forces, hydrophobic interactions, or hydrogen bonds among others, can ensure the formation of complexes. The types of interactions are important, not only because they determine the stability of the dendrimer-drug complexes, but they are also crucial to establish the kind of mechanism of uptake of the nanoformulations into the cell’s interior [73]. A cytotoxicity grade can occur depending on the type of interactions between the dendrimer and the different lipidic membranes. 

### 3.4. Dendrimer Interactions with Membranes

**Figure 2:**
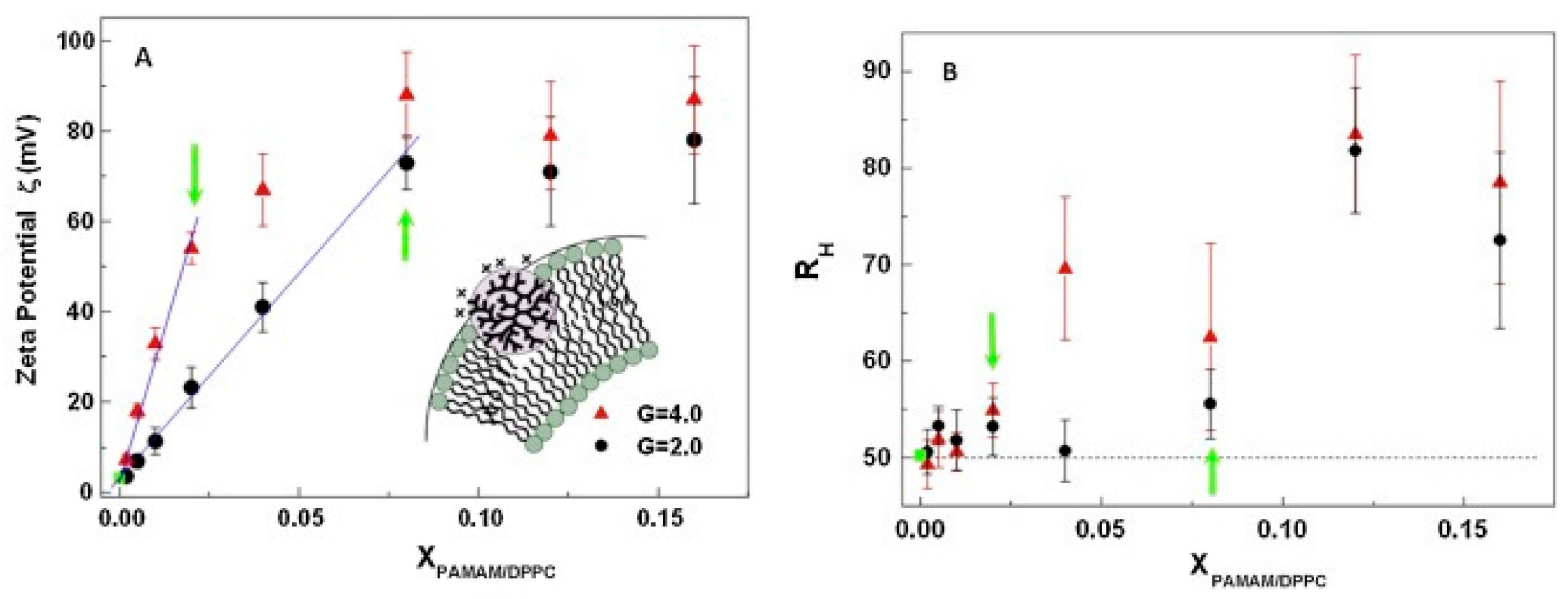
Measurements of: (**A**) Zeta potentials of extruded liposomes as a function of dendrimer-liposomes concentration, for G2-Poly(amidoamine) (PAMAM) and G4-PAMAM dendrimers. (**B**) Hydrodynamic radius of PAMAM-liposomes mixed system. This Figure was provided thanks to Reference [81].

As previously it has mentioned, the different kinds of interactions present between dendritic systems and membranes determine the success of the nanovehicles based on dendrimers [73]. The structural and organizational of biological membranes can vary depending on composition and charged lipids. Membrane lipids mainly phospholipids self-assemble form bilayers, where the hydrophobic interactions are main forces that support the membrane structures, which determine the thermodynamics of the system [80]. In addition, the membranes are also made up of integral or intrinsic proteins and peripheral proteins. The latter play an important role in various mechanisms, such as providing binding site for enzymes or signaling molecules. On the other hand, several sensitive techniques have been used to study the interactions between dendrimers and binding sites on the membranes, namely Z-potential, X-ray and neutron reflectivity, atomic force microscopy, ellipsometry, differential scanning calorimetry, and Raman spectroscopy, mainly. Recent studies about interactions of G2- and G4-PAMAM dendrimers and dipalmitoylphosphatidylcholine (DPPC) lipids evidenced that charged PAMAM dendrimers produce a perturbation due to penetration inside the DPPC vesicles alkyl chains by Raman scattering data [81]. The results show that the zeta potential of the vesicles increases linearly with the increase in the concentration of the positively charged dendrimer (Figure 2A). On the other hand, the hydrodynamic radius of DPPC liposomes at low dendrimer concentrations indicates the presence of pores with radius sizes comparable to those of membrane, (Figure 2B). Studies show that the dendrimer interacts with the DPPC liposome, both on the surface and in the hydrophobic region of the lipid bilayer. Such studies are crucial to propose dendrimers as attractive candidates for drug delivery systems. Those who must be to achieve biological targets such as tumor cells, since a solid tumor has a dense extracellular matrix, which restricts drug distribution within the tumor. 

**Scheme 5:**
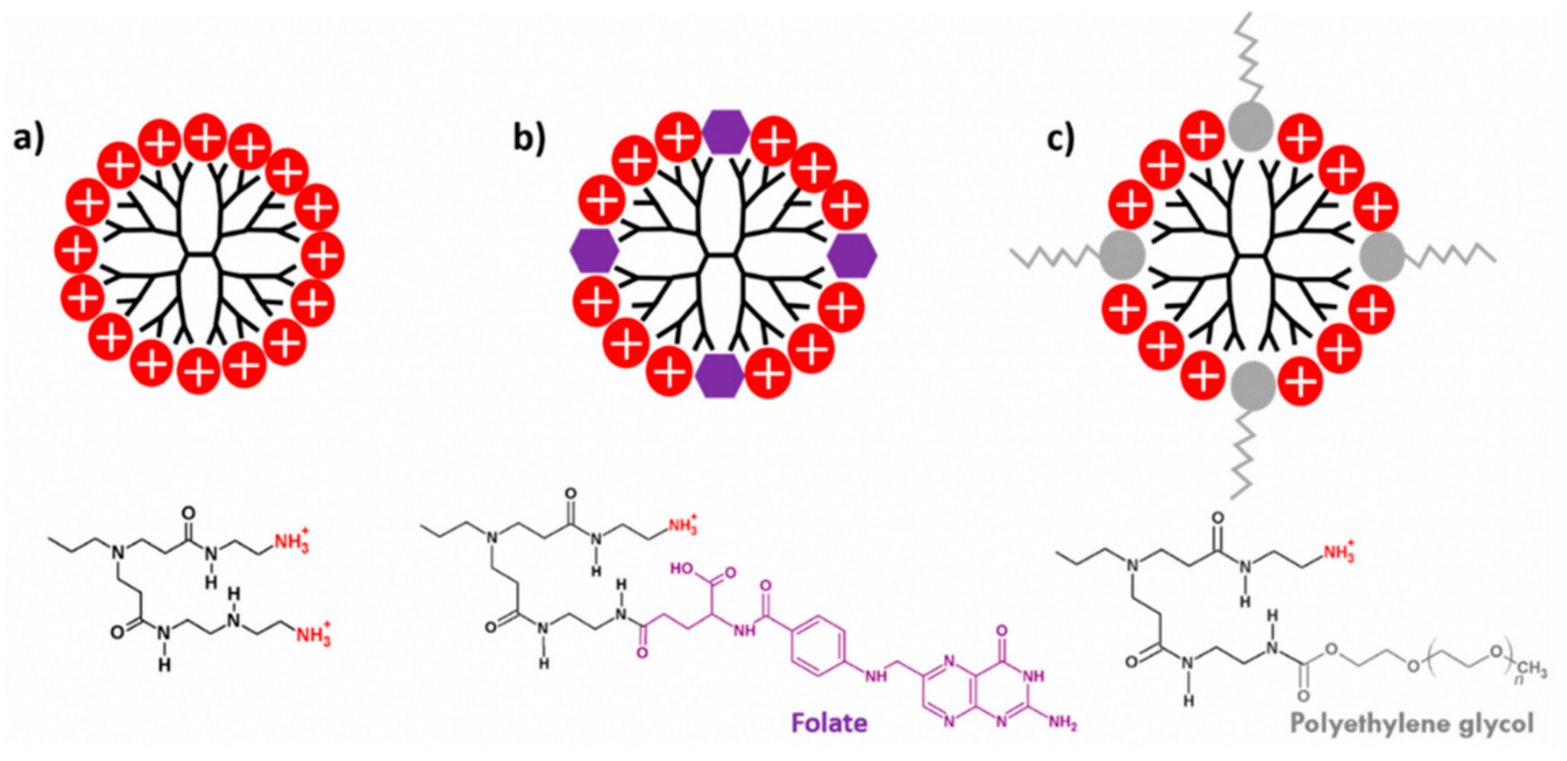
Schematic representation of G4-PAMAM functionalized with polyethylene glycol (PEG), and folate terminal groups: (**a**) G4-PAMAM dendrimer with positively charged superficial groups; (**b**) G4-PAMAM dendrimer functionalized 25% folate end-groups, and (**c**) G4-PAMAM dendrimer functionalized 25% PEG on the surface. This scheme was provided thanks to Reference [86].

In recent years, new strategies using nanoparticles to remedying the drug-resistant tumor cells, have been investigated [82,83,84]. The design of dendritic structures based on peptides functionalized with arginine have been used to cause a perturbing tumor membrane systems [85]. Arginine has been used to decorate the surface of dendrimers, since it can interact with the membrane lipids, through electrostatic interaction and hydrogen bonds. Furthermore, G4-PAMAM dendrimers functionalized with folate groups (FO) and polyethylene glycol (PEG) on the surface compare with G4-PAMAM without functionalizing were studied in hippocampal neurons [86] (Scheme 5). High cytotoxicity level was observed to G4-PAMAM in the cell viability assay. A reduction of toxic effect was observed to FO, while for PEG a better biocompatibility performance was shown. In addition, membrane permeability effects were carried to the same dendronized compounds, where G4-PAMAM showed an increased membrane permeability and loss of membrane integrity. Also, significant alterations caused by PFO and PEG in the capacitive currents during the test were not observed, indicating that the functionalization of G4 dendrimer is adequate to prevent effects on the membrane permeability [86].

## 4. Dendrimers to Drug Delivery Systems Applications

As mentioned earlier, thousands of reviews and articles have been published in the last decade on transport and contracted release of drugs by nanoparticles. These delivery systems have been of a relevant interest from industry to academic researches. The challenges in drug delivery systems are focused on solving the inefficient distribution of the drugs to the target tissues and the side effects improvement after drug administration that can influence both pharmacokinetic profiles as the biodistribution of them. The dendrimers, as we have said before, constitute a class of nanoparticles with physicochemical characteristics that make them very attractive in nanomedicine applications, more specifically as drug delivery systems (DDS). As previously mentioned, these extraordinary dendritic macromolecules have an improved membrane permeability and tumor retention effects [87,88,89], due to their suitable structural properties and size control. 

**Figure 3:**
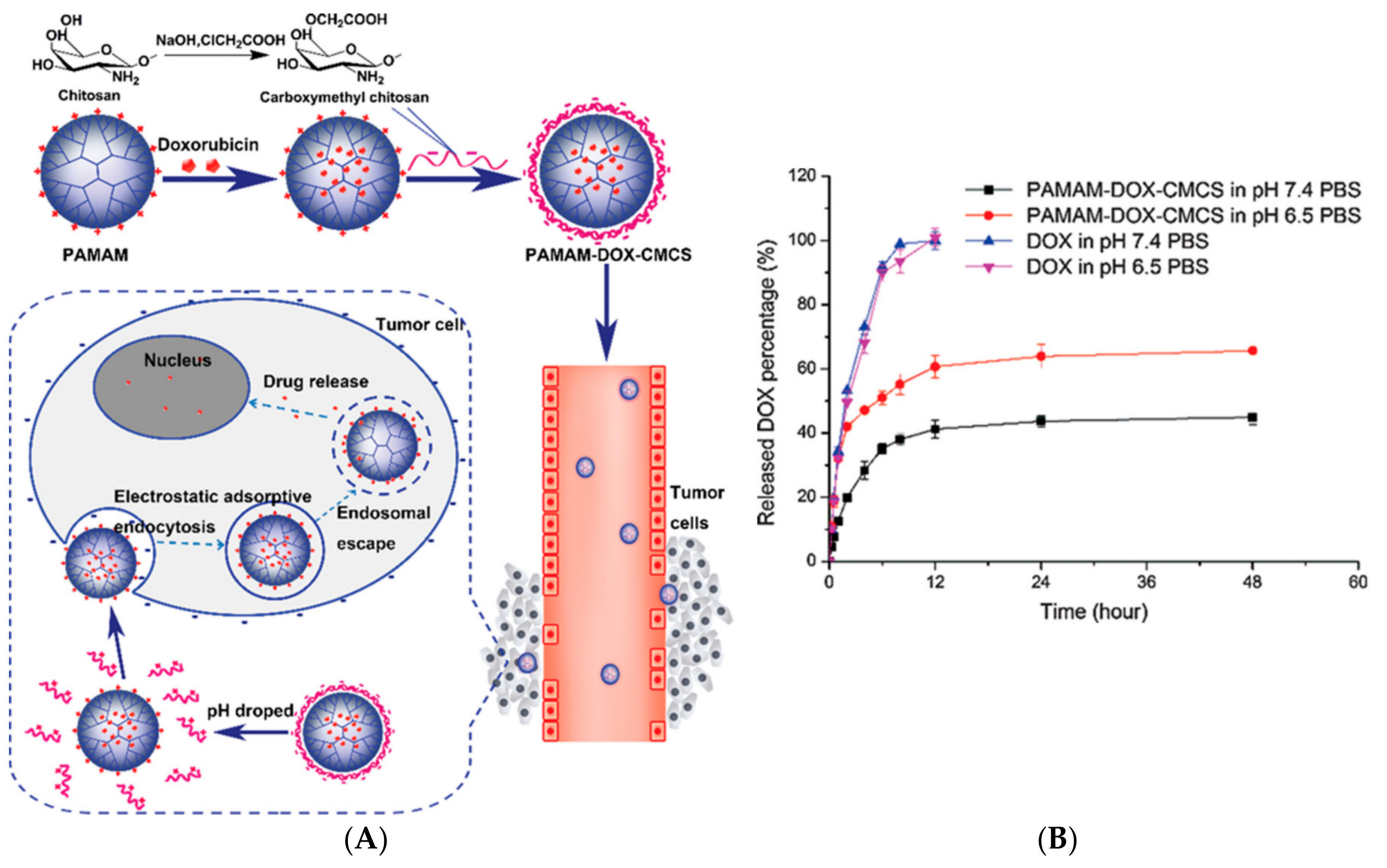
(**A**) Schematic representation of electrostatic conjugation of PAMAM dendrimers with carboxymethyl chitosan (CMCS) and the PAMAM-Doxorubicin-CMCS complex circulation through bloodstream and tumor microenvironment. (**B**) In vitro doxorubicin release with and without PAMAM-CMCS complex at pH 7.4 and pH 6.5. This Figure was provided thanks to Reference [90].

Dendrimers as DDS can improve key points in transport and biodistribution such as prolongation of the drug circulation time, enhance drug solubility, enhance tumor permeation and retention, protection of the drug from surroundings, and the ability to target diseased tissue, among others. As a strategy to minimize the cytotoxicity of PAMAM dendrimers, the positive surface charge of its surface has been modified with carboxymethyl chitosan (CMCS) [90,91,92]. Considering that, CMCS is an amphoteric polymer, sensitive pH, and taking doxorubicin as a model drug for cancer, it is that the formulation of a nanoparticle between PAMAM dendrimer and CMCS based on electrostatic conjugation, can transport the drug and release it into the target tissue (Figure 3A). It is known that there is a slight difference between physiological pH (7.4) and extracellular pH of tumor cells (6.5), (Figure 3B) so PAMAM dendrimers decorated with CMCS showed promising in vitro results of doxorubicin release at pH 6.5.

**Figure 4:**
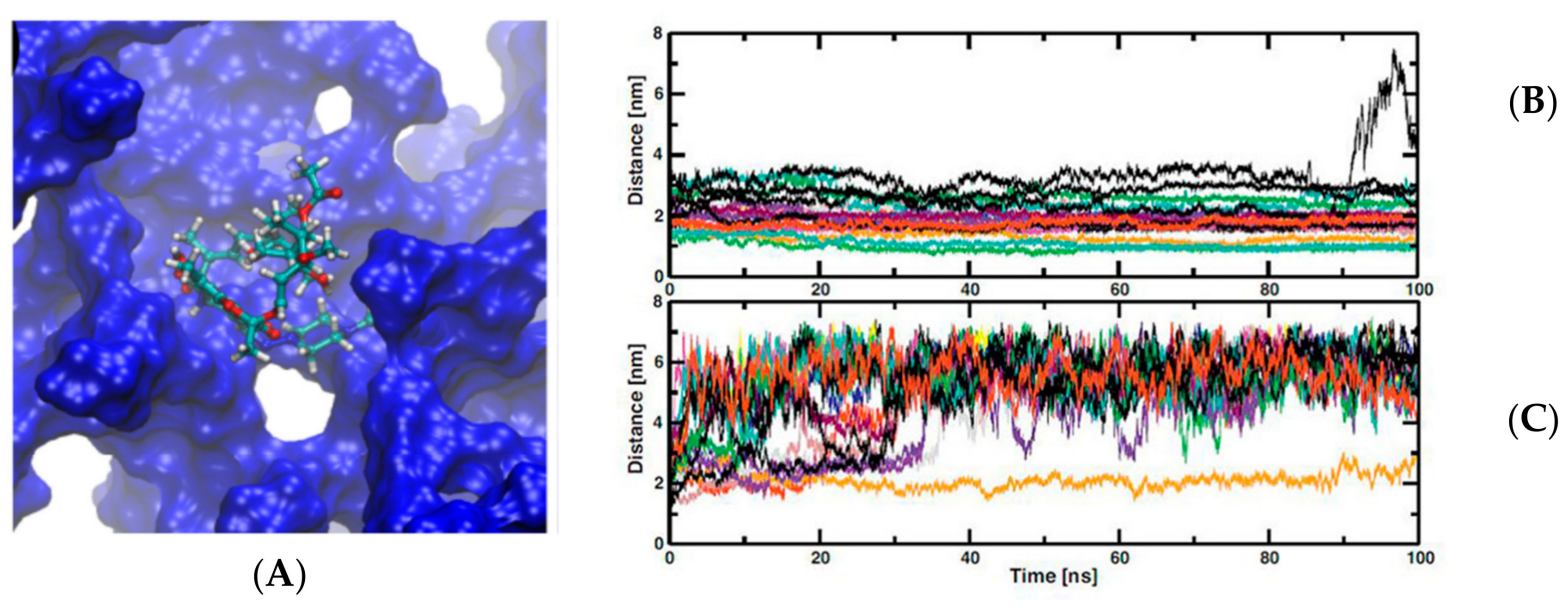
(**A**) a rifampicin (RIF) molecule inside one cavity of PAMAM dendrimer from molecular dynamic trajectory. (**B**,**C**) charts time of simulations against distance between center of mass of RIF molecules and dendrimer center of mass. (**B**) at neutral pH and (**C**) at low pH. This Figure was provided thanks to Reference [94].

Thus, dendrimer-drug conjugates used as pharmacological strategies can be decorated depending on the target tissue, the pathology of interest, and the type of drug to be transported. Otto et al. studied the solubility and dissolution properties of furosemide/PAMAM dendrimers complexes with different generations containing amino and ester terminal groups [91]. Pharmacokinetic assessments in vivo show that the dendrimer complexes improved the bioavailability of the drug compared to the free drug. Other drug reported with low solubility, rifampicin (RIF) an antibiotic to the tuberculosis treatment [93]*,* and important side effects since it suffers a hydrolysis reaction under gastric conditions was studied. Results of stability and drug-load capacity of the RIF-PAMAM complexes under different pH conditions were carried out by theoretical and experimental methods [94]. Studies demonstrated that twenty molecules of RIF per G4-PAMAM were determined according the results reported by molecular dynamic simulation tools. A theoretical study was carried out at two pH conditions, neutral and acid. At low pH, RIF molecules were quickly released to the solvent bulk, otherwise at neutral pH the RIF-PAMAM complex was more stable (Figure 4). Taking into account that drugs release strongly depends on the pH, may impose restrictions to administration way, namely in the case of oral administration due to the low pH of the stomach. Nevertheless, preliminaries studies have shown that PAMAM dendrimers have the potential for pulmonary inhalation, which may be advantageous in the case of respiratory diseases treatment [95,96]. These studies allow us determine what kind of dendrimer it is necessary to synthetize, according to the type of the target tissue and the type of drug will be transported.

### 4.1. Dendrimer as Drug Delivery Systems to Cancer Treatments

The different kinds of cancer that afflict the population are the leading cause of death worldwide. Despite the significant advances in medicine, there are still many challenges to be achieved in the treatment of cancer, to name a few, namely to decrease the side effects of some drugs [97,98], drug solubility improvement [76], drug-resistant cancer cell [84], and achieve a transport and targeted release of the drug [35]. Some of these advances focus on the use of dendritic nanoparticles as vehicles for the targeted transport of drugs against various types of cancer [85].

**Scheme 6:**
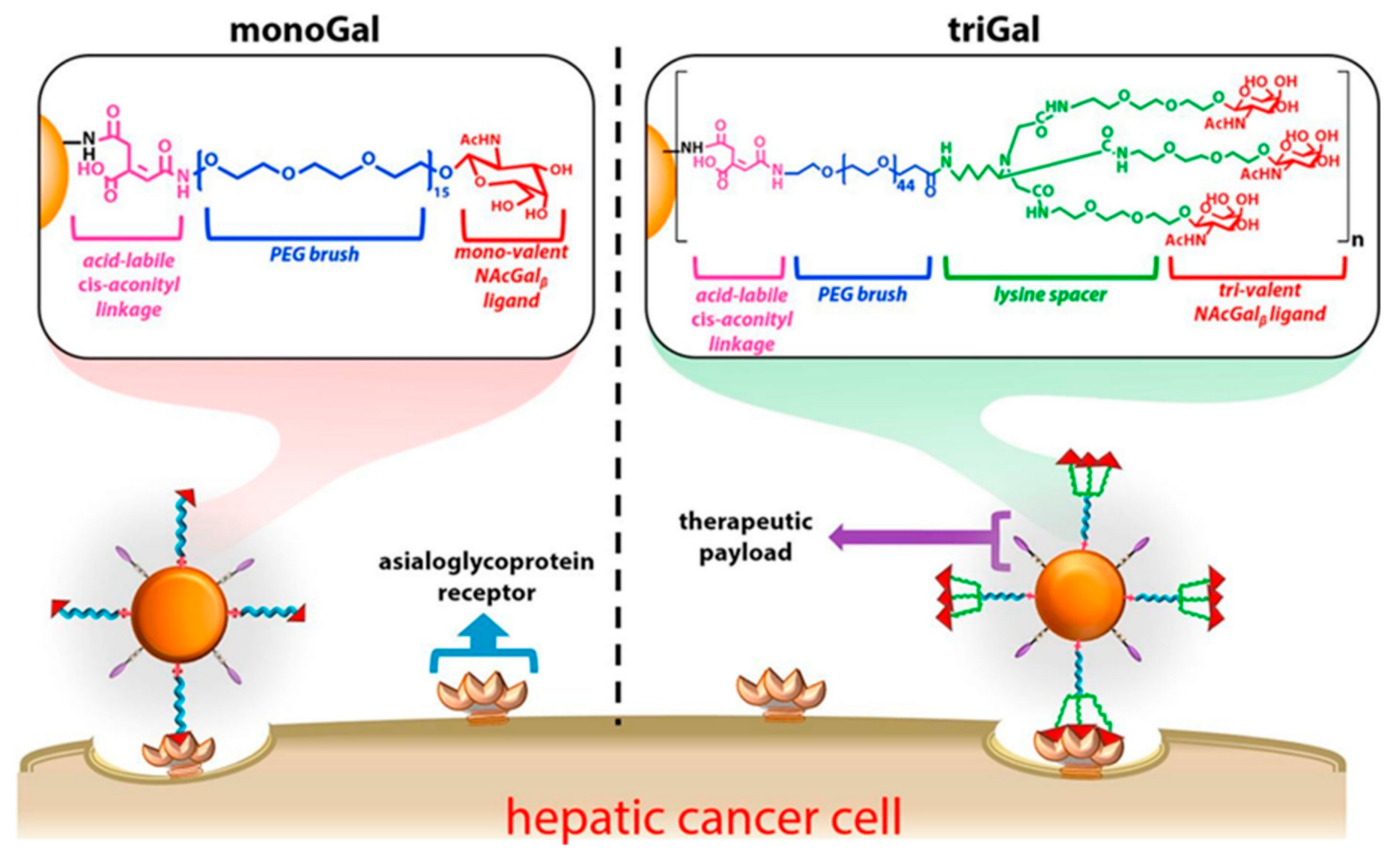
Schematic representation of G5-PAMAM dendrimer functionalized with polyethylene glycol (PEG brush) connecting to both nono-valent and tri-valent N-acetylgalactosamine (NAcGal), and folate as a linkage. This scheme was provided thanks to Reference [104].

Studies about hepatic cancer have determined that asialoglycoprotein receptor (ASGPR) is specifically overexpressed on tumor cells, and shown high binding affinity with glycoproteins [99,100]. The above can be considered as an advantage because key parts of the glycoproteins can be obtained and grafted on the surface of nanoparticles (NP) to promote high-efficiency binding to hepatic tumor cells. N-acetylgalactosamine (NAcGal) ligands on a NP surface achieves selective intake into hepatic cancer cells [101,102,103]. Moreover, Kurivilla et al. synthesized G5-dendrimers containing NAcGal ligands tri-valent (NAcGal3) attached to the surface through a PEG linker and measured their ability to achieve hepatic cancer cells in comparison to mono-valent ligands [104] (Scheme 6).

**Scheme 7:**
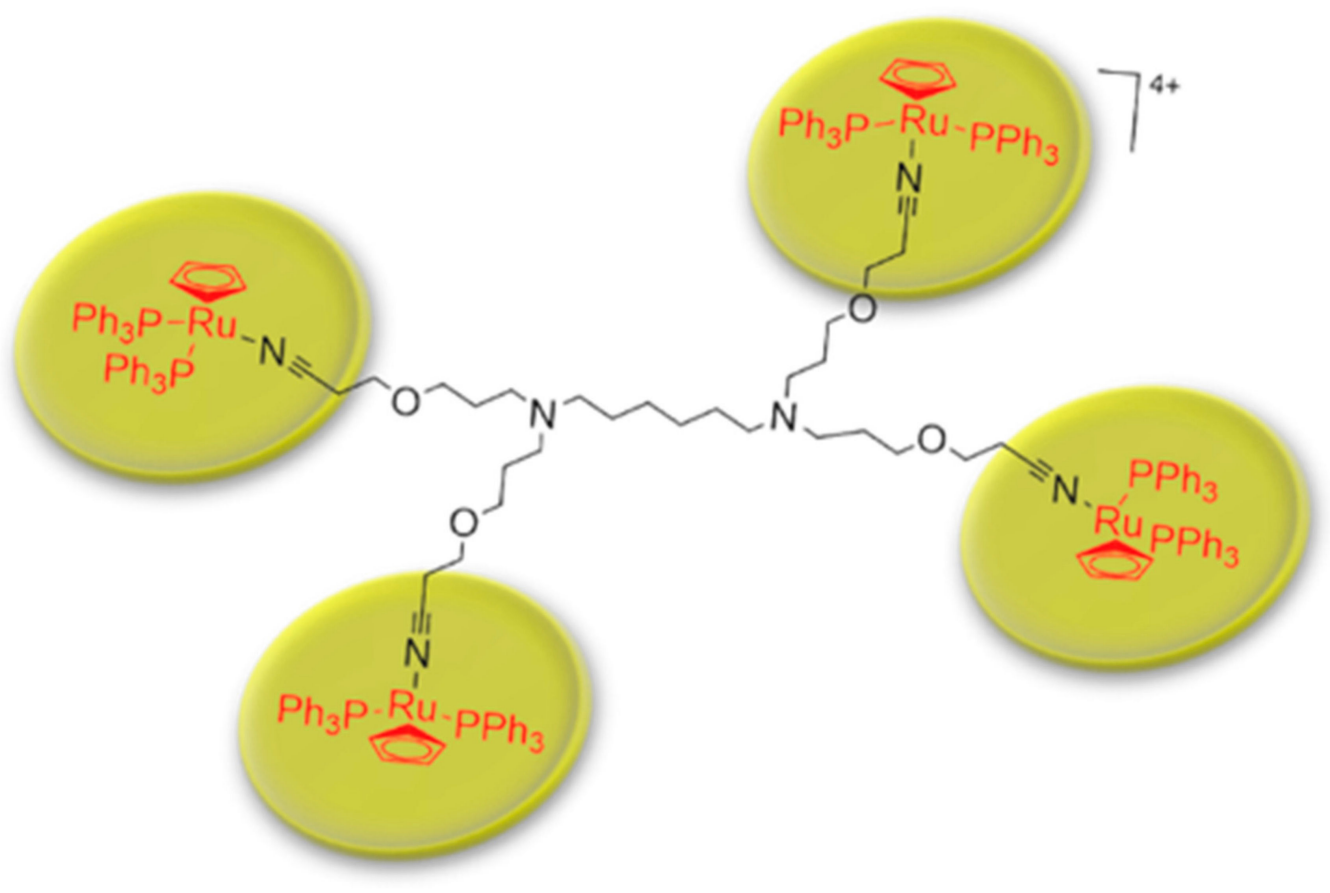
Schematic representation of metallodendrimer base on poly(alkylidenimine) dendrimer core containing nitrile groups and functionalized with [Ru(η^5^-C_5_H_5_)(PPh_3_)_2_Cl] compound. This scheme was provided thanks to Reference [105].

Metallodendrimers based on ruthenium to incorporate metals into dendritic scaffolds has been synthetize (Scheme 7) and characterize [105]. Several complexes based on ruthenium are in clinical phases against cancer therapies, however some complexes have had cytotoxicity problems. Evaluations of IC50 for metallodendrimers, organometallic complexes of ruthenium (Rucp) and cisplatin (cisPt) (a anticancer drug approval by FDA) in several of the carcinogenic cell lines were performed. The IC50 values for the metallodendrimers were the lowest compared to Rucp and cisPt. These results demonstrate that a lower concentration of metallodendrimer is needed to achieve 50% inhibition of cancer cell growth compared to Rucp and cisPt.

Several studies have shown that the direct administration of chemotherapeutic drugs for lung cancer significantly improves the exposure and residence of the drug in comparison with intravenous administration treatments. PEGylated polylysine dendrimers, conjugated to doxorubicin (DOX) to promote the controlled and prolonged exposure of lung-resident cancer to the cytotoxic drug, have been studied. The results show that PEGylated polylysine dendrimers have great potential as inhalable chemotherapeutic nanoformulations, improving the exposure of lung tumor to a cytotoxic drug [106]. Likewise, conjugates of DOX linked to PEGylated G4-polylysine dendrimer were studied to determine drug delivery kinetics, intravenous, and pulmonary pharmacokinetics in rats [107]. Cathepsin B-cleavable peptides were used to drug-linker since the extracellular and lysosomal expression of this enzyme is highly upregulated by cancer cells.

**Scheme 8:**
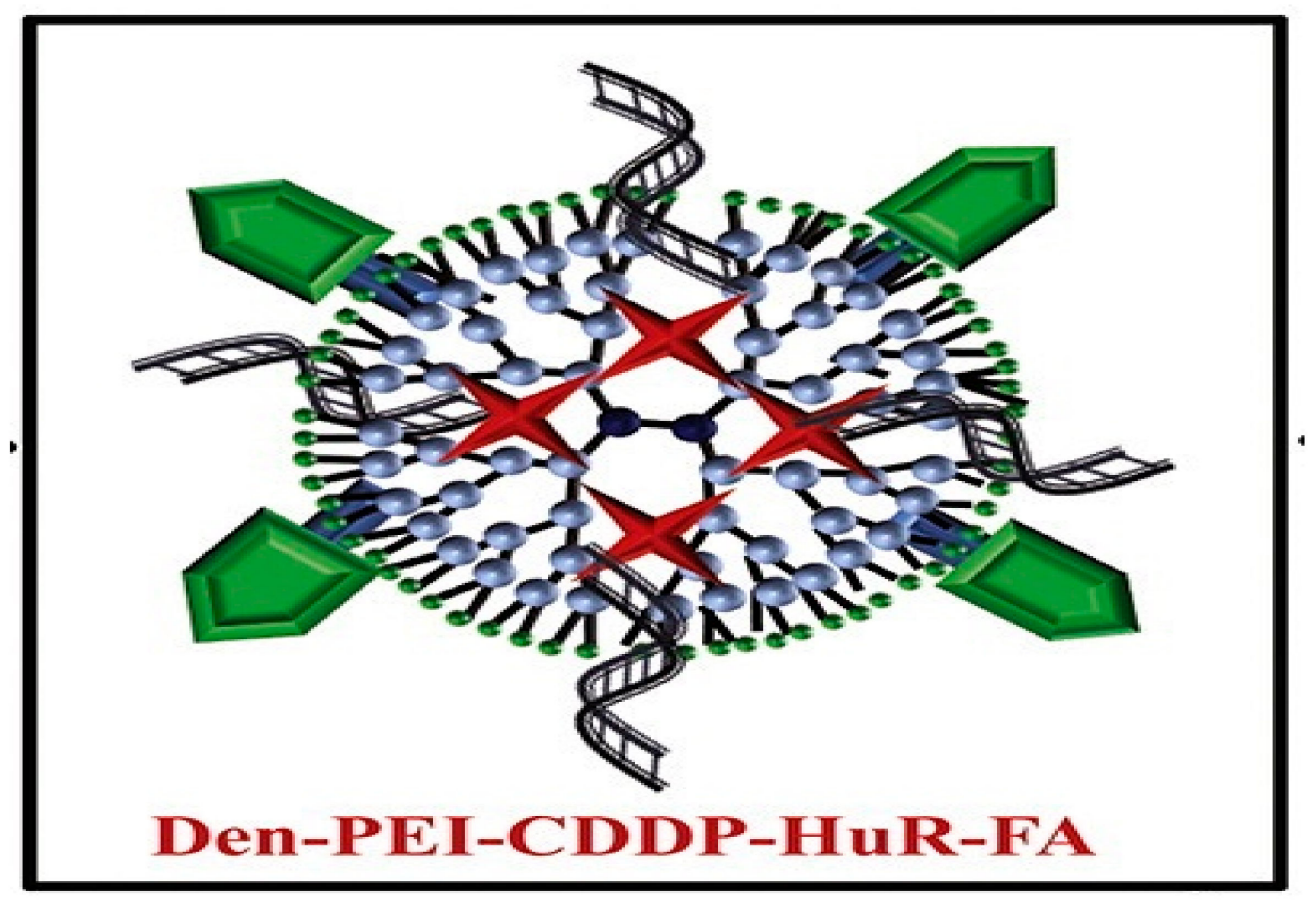
Therapeutic nanoformulation based on polyethyleneimine dendrimer functionalized with folic acid (FA), human antigen R (HuR), and cis-diamine platinum (CDDP). This scheme was provided thanks to Reference [109].

Cis-diamminodichloridoplatinum (II) (CDDP) is a anticancer drug used for the treatment of lung cancer. This drug intercalates into the cellular DNA, forming DNA adducts resulting in apoptosis [108]. A novel strategy chemotherapeutic combination for lung cancer was developed based on a folic acid (FA) conjugated polyamidoamine dendrimer. This formulation was proposed for co-delivery of siRNA against human antigen R and cis-diamine platinum (CDDP) to folate receptor-α (FRA) overexpressing (H1299) lung cancer cell [109] (Scheme 8). Studies reveal that folic acid-conjugated dendrimers generate considerable DNA damage and apoptosis cell death compared to non-functionalized nanoparticles with FA.

While positively charged dendrimers possess considerable cellular cytotoxicity, other strategies have been proposed to formulate dendritic nanoparticles. The formulation of dendrimers negatively charged on the surface has been proposed as a way to increase the viability of healthy tissues. Negatively charged poly(amido amine)-2,3-dimethylmaleic monoamine (PAMAM-DMA) dendrimers were prepared by Cao et al. [110], which possess the capacity to change their load in response to acid pH, present in a tumor environment. Low cytotoxicity in the normal/neutral environment was observed for negatively charged PAMAM-DMA dendrimers. Co-administration of DOX plus G5-PAMAM-DMA in mice bearing MCF-7 tumors enhanced the efficacy of tumor growth inhibition compared with the administration of free DOX.

Li et al. [111] has developed a novel and practical photothermal hydrogel, based on platinum nanoparticles encapsulated with dendrimer (DEPts) dextran. Photothermal hydrogel allowed repeating the photothermal therapy (PTT) and reduced the toxicity induced by long-term retention. The hydrogel represented an excellent photothermal effect and excellent biocompatibility. It was able to remain in tumors for days to allow repeated PTT, leading to complete tumor regression. Additionally, anti-Flt1 antibody-conjugated polyethylene glycol (PEG)-cored poly(amidoamine) (PAMAM) dendrimers improve the effectiveness of the gemcitabine against pancreatic cancer. Tissues, such as the liver and the bone marrow, which are known to have high vascular endothelial growth factor (VEGF)–Flt1 pathway activity, were targeted by gemcitabine when delivered through anti-Flt1 antibody-conjugated PAMAM dendrimers [112]. The advantage of using chemotherapeutic agents complexed with dendrimers not only improve anticancer efficacy but also assist in the elimination of the tumor-induced myeloid cells.

Undoubtedly it can be said that transport systems for cancer drugs based on dendrimers are the most studied and published [113,114,115,116,117,118]. This high interest is due to its versatility to mold them to the need of the system under study. A large number of works every year are published on these drug nanocarriers.

### 4.2. Dendrimer as Drug Delivery Systems to Cardiovascular Treatments

Cardiovascular diseases (CVDs) remains the leading cause of morbidity and mortality around the world. CVDs include diseases to heart, vascular diseases of brain and diseases of blood vessels, and are responsible for over 17.3 million deaths per year [119]. This pathology is associated with the upregulation of inflammatory genes. Gene silencing using RNA interference is a technic to regulate gene expression in CVDs, but the lack of efficient delivery systems has prevented its correct application. An important hormonal system involved in CVDs is the Renin-Angiotensin-Aldosterone System (RAAS). Angiotensin II (Ang II), a peptide of eight amino acids regulates the main effects of RAAS [120]. Therefore, overexpression of Ang II can cause a series of complications that can lead to heart failure. Due to these reasons, the inhibition of Ang II activation is an objective within the therapies for CVDs [121]. Liu et al. formulated a nanocarrier to complex siRNA and a cell penetration peptide (CPP) that allows improving the internalization of complexed siRNA within cardiomyocytes [122]. PEG segment was included in the structure to reduce the PAMAM toxicity. The results display that CPP conjugated with dendrimer was non-toxic and efficient to the siRNA delivery system. Bioinformatic analysis showed that the molar ratio of union between Ang-(1-7) and PAMAM-OH dendrimer as 2:1. Molecular dynamics simulation analysis revealed that the ability of neutral PAMAM-OH to protect Ang-(1-7) and form stable complexes. In short, the complex Ang-(1-7)/PAMAM-OH is an efficient administration method for Ang-(1-7), since it improves the anti-atrophic activity of this peptide in skeletal [123].

Myocardial ischemia can be addressed through gene therapy of vascular endothelial growth factor (VEGF) to promote therapeutic angiogenesis. However, the unregulated expression of VEGF and the use of viral vectors have stopped angiogenic therapy. Won et al. developed and evaluated a bioreducible polymer dendrimer-type, PAM-ABP to conjugate with a VEGF plasmid, pb-SP-ODD-VEGF [124]. This complex base on a dendrimer shows great potential as a therapy for the treatment of myocardial ischemia and infarction.

## 5. Conclusions

Dendrimers are multifunctional macromolecules that can be used in several fields. The nano-size, tunable surface, interaction with cell membranes, interaction with drugs, interior cavities, among other features, make dendrimers excellent candidates for drug delivery systems (DDS). Due to their chemical versatility, dendrimers have been applied to the transport of various types of bioactive molecules, namely drug-type molecules and genes. In the last decade, the use of dendrimer as DDS has been reported approximately one thousand publications, according to the WOS database. Mainly, the highest number of researches in the area of DDS using dendrimers has been for cancer diseases. A large number of studies reported for lung cancer involving dendrimers have been reported. In past years, the DDS for lung cancer has combined strategies, conjugating drugs in dendrimers, as well as gene agents for cell recognition. In addition, dendrimers are being studied as DDS in cardiovascular diseases. Due to low bioavailability and low cellular transfection of some bioactive molecules, formulations have been prepared to inhibit agents that affect cardiovascular pathologies, e.g., angiotensin has been conjugated with dendrimers to form stable complexes [125]. The controlled release of therapeutic agents and decreasing of side effects are challenges that are continuously being addressed by researchers. In conclusion, dendrimers are amazing macromolecular platforms that can be modified depending on the chemical nature of the drug that will be transported and also the target tissue. The physicochemical properties of dendrimers are important to study to understand the transport and release of drugs, as well as internalization and intracellular traffic.

## Figures and Tables

**Table 1:** Summary of some synthetic strategies and dendrimers synthesized.

Synthesis Strategy	Dendrimer Type	Authors
Divergent	Sulfonimide-based dendrimers	Lukin et al. [38]
	Tetraphenylmethane-ethynylene dendrimer	Julio I. Urzúa et al. [39]
	Rhodamine B dendrimers	Lyu et al. [37]
	Porphyrin and metalloporphyrin dendrimers	Alsehli et al. [40]
	Poly(amidoamine)(PAMAM) dendrimer-grafted graphene oxide	Gholipour-Mahmoudalilou et al. [41]
	Benzylic viologen dendrimers	Kathiresan et al. [42]
	Newkome-type polyamide-based dendrimers	Kaufman et al. [43]
	Phosphorus containing dendrimer	Dadapeer et al. [44]
Convergent	G2-PEG dendrimer	Patil et al. [45]
	dodecaruthenium organometallic dendrimer	Merhi et al. [46]
	Azobenzene core dendrimer with Chalcone motif	Selvarani et al. [47]
	poly(amino-ester) dendrons	Dayyani et al. [48]
	2,2-bis-(hydroxymethyl)-propionic acid (bis-MPA) hyperbranched linear dendrimers	Guizzardi et al. [49]
	mesitylene-based polyamine dendrimer	Singh et al. [50]
	poly ɛ-caprolactone linear block, poly(amino-ester) dendritic block and m-PEG linear block	Khoee et al. [51]
Click chemistry	Glycopeptide dendrimers based on β-cyclodextrin	Pu-Guang et al. [52]
	poly(thioglycerol-2-propionate) dendrimers	Fan et al. [53]
	carbazole core diblock codendrimers	Han et al. [54]
